# Supplementary material for: Modeling glioblastoma heterogeneity as a dynamic network of cell states
Source: Mol Syst Biol. 2021 Sep 16;17(9):e10105. doi: 10.15252/msb.202010105 (PMC8444284; doi:10.15252/msb.202010105)
Supplement: Supplementary file 6 — Source Data for Figure 5 [file MSB-17-e10105-s004.zip › Figure5A_sourcedata/GSEA_3071/nosigdb_stateII.GseaPreranked.1622795151171/RICHARDS_VERHAAK_CANCERCELL_2010_PRONEURAL.html]

Details for gene set RICHARDS\_VERHAAK\_CANCERCELL\_2010\_PRONEURAL[GSEA]

|  || Dataset | state23071 |
| Phenotype | NoPhenotypeAvailable |
| Upregulated in class | na\_neg |
| GeneSet | RICHARDS\_VERHAAK\_CANCERCELL\_2010\_PRONEURAL |
| Enrichment Score (ES) | -0.60832256 |
| Normalized Enrichment Score (NES) | -3.1034484 |
| Nominal p-value | 0.0 |
| FDR q-value | 0.0 |
| FWER p-Value | 0.0 |
Table: GSEA Results Summary

  

Fig 1: Enrichment plot: RICHARDS\_VERHAAK\_CANCERCELL\_2010\_PRONEURAL      
 Profile of the Running ES Score & Positions of GeneSet Members on the Rank Ordered List

  

| PROBE | GENE SYMBOL | GENE\_TITLE | RANK IN GENE LIST | RANK METRIC SCORE | RUNNING ES | CORE ENRICHMENT || 1 | MCM10 |  |  | 9 | 1.654 | 0.0266 | No |
| 2 | ATAD5 |  |  | 18 | 1.469 | 0.0503 | No |
| 3 | ALCAM |  |  | 288 | 0.771 | -0.0030 | No |
| 4 | CDC25A |  |  | 572 | 0.589 | -0.0629 | No |
| 5 | FERMT1 |  |  | 965 | 0.454 | -0.1523 | No |
| 6 | CDC7 |  |  | 971 | 0.451 | -0.1457 | No |
| 7 | DPF1 |  |  | 1169 | 0.403 | -0.1875 | No |
| 8 | STMN1 |  |  | 1249 | 0.387 | -0.2004 | No |
| 9 | TOPBP1 |  |  | 1355 | 0.366 | -0.2200 | No |
| 10 | P2RX7 |  |  | 1383 | 0.360 | -0.2204 | No |
| 11 | TAF5 |  |  | 1869 | 0.290 | -0.3357 | No |
| 12 | E2F3 |  |  | 1961 | 0.277 | -0.3535 | No |
| 13 | CBX1 |  |  | 2210 | 0.251 | -0.4106 | No |
| 14 | ATAT1 |  |  | 2258 | -0.255 | -0.4179 | No |
| 15 | TMEFF1 |  |  | 2448 | -0.277 | -0.4599 | No |
| 16 | OLIG2 |  |  | 2453 | -0.278 | -0.4561 | No |
| 17 | SRGAP3 |  |  | 2508 | -0.286 | -0.4645 | No |
| 18 | CLASP2 |  |  | 2514 | -0.287 | -0.4607 | No |
| 19 | YPEL1 |  |  | 2589 | -0.299 | -0.4738 | No |
| 20 | BCL7A |  |  | 2603 | -0.301 | -0.4718 | No |
| 21 | GSK3B |  |  | 2641 | -0.307 | -0.4756 | No |
| 22 | GNG4 |  |  | 2702 | -0.316 | -0.4850 | No |
| 23 | MLLT11 |  |  | 2760 | -0.328 | -0.4934 | No |
| 24 | ZNF821 |  |  | 2776 | -0.331 | -0.4914 | No |
| 25 | PAK3 |  |  | 2839 | -0.343 | -0.5008 | No |
| 26 | ZNF711 |  |  | 2953 | -0.365 | -0.5224 | No |
| 27 | ZNF286A |  |  | 2986 | -0.370 | -0.5239 | No |
| 28 | ABAT |  |  | 3075 | -0.391 | -0.5389 | No |
| 29 | FAM110B |  |  | 3191 | -0.426 | -0.5600 | No |
| 30 | ARHGEF9 |  |  | 3270 | -0.446 | -0.5716 | No |
| 31 | DNM3 |  |  | 3290 | -0.454 | -0.5684 | No |
| 32 | GSTA4 |  |  | 3318 | -0.463 | -0.5670 | No |
| 33 | MARCKS |  |  | 3369 | -0.487 | -0.5709 | No |
| 34 | LRP6 |  |  | 3473 | -0.530 | -0.5872 | No |
| 35 | FHOD3 |  |  | 3559 | -0.576 | -0.5983 | Yes |
| 36 | ERBB3 |  |  | 3561 | -0.578 | -0.5884 | Yes |
| 37 | CRMP1 |  |  | 3583 | -0.594 | -0.5833 | Yes |
| 38 | PODXL2 |  |  | 3599 | -0.604 | -0.5765 | Yes |
| 39 | MPPED2 |  |  | 3701 | -0.685 | -0.5896 | Yes |
| 40 | SATB1 |  |  | 3776 | -0.767 | -0.5946 | Yes |
| 41 | TOP2B |  |  | 3777 | -0.767 | -0.5812 | Yes |
| 42 | RUFY3 |  |  | 3785 | -0.773 | -0.5694 | Yes |
| 43 | GRIA2 |  |  | 3786 | -0.774 | -0.5559 | Yes |
| 44 | SOX4 |  |  | 3797 | -0.786 | -0.5447 | Yes |
| 45 | TTYH1 |  |  | 3807 | -0.795 | -0.5330 | Yes |
| 46 | CDKN1B |  |  | 3812 | -0.800 | -0.5201 | Yes |
| 47 | C1QL1 |  |  | 3828 | -0.819 | -0.5095 | Yes |
| 48 | PFN2 |  |  | 3841 | -0.832 | -0.4980 | Yes |
| 49 | MARCKSL1 |  |  | 3852 | -0.845 | -0.4857 | Yes |
| 50 | ZEB2 |  |  | 3876 | -0.894 | -0.4758 | Yes |
| 51 | CHD7 |  |  | 3880 | -0.904 | -0.4608 | Yes |
| 52 | DLL3 |  |  | 3905 | -0.956 | -0.4500 | Yes |
| 53 | NRXN2 |  |  | 3907 | -0.961 | -0.4335 | Yes |
| 54 | SOX2 |  |  | 3953 | -1.069 | -0.4260 | Yes |
| 55 | NCAM1 |  |  | 3966 | -1.106 | -0.4097 | Yes |
| 56 | SOX11 |  |  | 3977 | -1.147 | -0.3922 | Yes |
| 57 | TMCC1 |  |  | 3980 | -1.161 | -0.3724 | Yes |
| 58 | MMP16 |  |  | 3983 | -1.171 | -0.3524 | Yes |
| 59 | MYO10 |  |  | 3990 | -1.193 | -0.3331 | Yes |
| 60 | GPM6A |  |  | 4002 | -1.248 | -0.3141 | Yes |
| 61 | SLCO5A1 |  |  | 4015 | -1.362 | -0.2933 | Yes |
| 62 | MAP2 |  |  | 4051 | -1.673 | -0.2728 | Yes |
| 63 | ASCL1 |  |  | 4061 | -1.762 | -0.2442 | Yes |
| 64 | BEX1 |  |  | 4072 | -2.034 | -0.2112 | Yes |
| 65 | CKB |  |  | 4075 | -2.149 | -0.1742 | Yes |
| 66 | C1orf61 |  |  | 4084 | -2.474 | -0.1330 | Yes |
| 67 | DGKI |  |  | 4085 | -2.502 | -0.0894 | Yes |
| 68 | FXYD6 |  |  | 4089 | -2.606 | -0.0446 | Yes |
| 69 | DCX |  |  | 4091 | -2.671 | 0.0017 | Yes |
Table: GSEA details [plain text format]

  

Fig 2: RICHARDS\_VERHAAK\_CANCERCELL\_2010\_PRONEURAL: Random ES distribution      
 Gene set null distribution of ES for **RICHARDS\_VERHAAK\_CANCERCELL\_2010\_PRONEURAL**
